# A new experimental phenomenological method to explore the subjective features of psychological phenomena: its application to binocular rivalry

**DOI:** 10.1093/nc/niaa018

**Published:** 2020-10-03

**Authors:** Takuya Niikawa, Katsunori Miyahara, Hiro Taiyo Hamada, Satoshi Nishida

**Affiliations:** n1 Institut Jean Nicod, Ecole normale supérieure, Paris, France; n2 Faculty of Humanities and Human Sciences, Hokkaido University, Sapporo, Japan; n3 School of Liberal Arts, University of Wollongong, Wollongong, Australia; n4 Graduate School of Arts and Sciences, University of Tokyo, Tokyo, Japan; n5 Autonomous Agent Team, Araya Inc., Minato-ku, Tokyo, Japan; n6 Department of Functional Brain Imaging Research, National Institute of Radiological Sciences, National Institutes for Quantum and Radiological Science and Technology, Inage-ku, Chiba, Japan; n7 Neural Computation Unit, Okinawa Institute of Science and Technology, Onna, Okinawa, Japan; n8 Center for Information and Neural Networks (CiNet), National Institute of Information and Communications Technology (NICT), Suita, Osaka, Japan; n9 Graduate School of Frontier Biosciences, Osaka University, Suita, Osaka, Japan

**Keywords:** binocular rivalry, consciousness, phenomenology, experimental phenomenology, phenomenological method

## Abstract

The subjective features of psychological phenomena have been studied intensively in experimental science in recent years. Although various methods have been proposed to identify subjective features of psychological phenomena, there are elusive subjective features such as the spatiotemporal structure of experience, which are difficult to capture without some additional methodological tools. We propose a new experimental method to address this challenge, which we call the *contrast-based experimental phenomenological method* (CEP). CEP proceeds in four steps: (i) front-loading phenomenology, (ii) online second-personal interview, (iii) questionnaire survey, and (iv) hypotheses testing. It differs from other experimental phenomenological methods in that it takes advantage of *phenomenal contrasts* in collecting phenomenological data. In this paper, we verify the validity and productivity of this method by applying it to binocular rivalry (BR). The study contributes to empirical research on BR in three respects. First, it provides additional evidence for existing propositions about the subjective features of BR: e.g. the proposition that the temporal dynamics of the experience depend upon subject-dependent parameters such as attentional change. Second, it deepens our understanding of the spatiotemporal structures of the transition phase of BR. Third, it elicits new research questions about depth experience and individual differences in BR. The presence of such contributions demonstrates the validity and productivity of CEP.

**Highlights**

- We propose a new experimental method to explore the subjective features of psychological phenomena.
- We apply the method to binocular rivalry (BR) to demonstrate its validity and productivity.
- The method contributes to deepening and extending the understanding of the subjective aspects of BR.

## Introduction

Cognitive science typically conceptualizes psychological phenomena in terms of cognitive functions and seeks to explain them in terms of computational and neural mechanisms (Thagard 2019). However, many psychological phenomena also have *subjective features* that are not immediately captured in functionalist terms. For instance, emotion is often characterized in terms of a set of functions to use rewards and punishers to guide behavior (Rolls 2013, chap. 3). But it is questionable whether the nature of emotion can be fully captured by those functions, for there is also something it is like to have emotions (such as the tangible feeling of being sad, happy, or angry) (Nagel 1974). Any complete theory of a psychological phenomenon should develop cognitive and neural models that do not only account for their cognitive functions but also accommodate their subjective features.

This gives rise to a methodological question: How can objective science identify the subjective features of experience? One simple method is to ask one to report what they experience. For instance, we can determine whether one sees a house or a face by asking them to press a button to report which they experience (Tong *et al.* 1998) or whether one is aware of a change in a screen by asking them to verbally report any change of which they are aware (Rensink *et al.* 1997). Another method is to instruct participants to carry out a task from which we can infer what experience they have. For instance, we can determine when one experiences two patches as having the same color by instructing to control the color of one patch so that it matches the other patch (Sarkar *et al.* 2010) or can scale some aspects of one’s sensory experience, such as brightness and loudness, by instructing to assign numbers to represent their magnitudes (Teghtsoonian 2015). These methods work very well for subjective features of experience that are fragmentally extracted from the whole experience (e.g. a change in a screen or the hue of color patches). However, it is difficult to apply them directly to more elusive features that are not readily available to introspective observation (e.g. holistic features of the spatiotemporal structure of experience). This suggests that additional methods are required to uncover these more elusive subjective features of experience.

Phenomenology is a philosophical tradition that is centrally concerned with systematically exploring such elusive features of consciousness. In recent years, researchers have proposed various methods that combine phenomenology and experimental psychology to expand the scope of scientific psychology (for a concise overview of experimental phenomenological methods, see Miyahara *et al.* 2020). For example, some propose to train experimental participants in skills to reflect on and describe their experience, which will allow experimenters to obtain more sophisticated subjective reports (Varela 1996; Lutz 2002; Miyahara *et al.* 2020). Another proposed method is to interview experimental participants systematically about their experience (Petitmengin 2006; Valenzuela Moguillansky *et al.* 2013). Alternatively, some have developed detailed questionnaires designed to capture the subjective features of target experiences (Pekala 1991; Studerus *et al.* 2010). Nevertheless, it is fair to say that experimental phenomenology is still a very rudimentary domain of inquiry, requiring much methodological improvement.

In this paper, we propose a novel experimental schema to be added to the toolbox of experimental phenomenology. The proposed schema consists of the following four steps: (i) *Front-loading phenomenology*: Formulate hypotheses about the subjective features of the target psychological phenomenon; (ii) *Online second-personal interview*: Conduct a face-to-face interview to participants who are undergoing the psychological phenomenon in question in order to collect descriptions of its subjective features; (iii) *Questionnaire survey*: Obtain responses to a questionnaire designed to test said hypotheses from the participants; (iv) *Hypothesis-testing*: Evaluate said hypotheses based on the data acquired through the second and third steps.

A distinctive feature of our experimental schema is that it creatively incorporates the method of *phenomenal contrast*. Some philosophers have suggested that disputes about subjective features, which cannot be settled in terms of direct introspection of the psychological phenomenon at issue, may be resolved by comparing it with similar phenomena (Kriegel 2007; Siegel 2007). The idea is that the subjective features of a psychological phenomenon that are hard to notice by simple introspection can be brought into relief by contrasting it with similar psychological phenomena. Our proposal employs the phenomenal contrast method in the second and third steps of the experimental schema described above, namely the data collection processes. The online second-personal interview is designed to make the participants describe the subjective features of a target psychological phenomenon *in comparison to a similar yet different kind of experience*. The questionnaire is also designed to take advantage of the comparison. This focus on phenomenal contrast makes our experimental schema distinct from other phenomenological/qualitative psychological approaches such as microphenomenology (Petitmengin 2006; Petitmengin *et al.* 2019; Valenzuela-Moguillansky and Vásquez-Rosati 2019) and experience sampling methods (Larson and Csikszentmihalyi 1983; Hurlburt and Akhter 2006). Furthermore, our experimental schema can be easily incorporated into a standard psychophysical framework in which two psychological conditions with different target variables are put in contrast (e.g. tracking a visual target with full attention and with diverted attention). We call our experimental schema *the contrast-based experimental phenomenological method* (CEP).

The objective of this paper is to demonstrate the validity and productivity of CEP. The validity of CEP is questioned if it delivers results that are in conflict with robust existing findings about the subjective features of a target psychological phenomenon. The productivity of CEP is questioned if it fails to advance existing research on a psychological phenomenon in any direction. CEP is thus considered to be valid and productive if it delivers broadly consistent results with robust existing findings about a target psychological phenomenon and provide new understanding and insights about its subjective features.

To test the validity and productivity of CEP, we apply it to explore the subjective features of *binocular rivalry* (BR). In BR, participants are presented with different monocular stimuli to each of their eyes. Then instead of seeing a stable mixed image of the two stimuli, they have a visual experience of ongoing alternations between the two images. BR has recently attracted much attention in cognitive neuroscience, in particular, consciousness studies (Miller 2013; Blake *et al.* 2014; Wu 2018, sec. 5.2).

There is a fair amount of studies that examine the subjective features of BR. The subjective aspect of BR has typically been characterized in terms of *the dominance of each image* and *its alternation*. Many pieces of research have explored how the dominance duration and the alternation rate change depending on the types of stimuli and other variables (Fahle 1982; Blake *et al.* 1992; Carter *et al.* 2007; Pelekanos *et al.* 2012; Brascamp *et al.* 2015; Paris *et al.* 2017; Skerswetat *et al.* 2018). In particular, many have focused on the influence of attention on the dominance duration and the alternation rate (Ooi and He 1999; Paffen and Van der Stigchel 2010; Brascamp and Blake 2012; Moreno-Sánchez *et al.* 2019). However, the subjective aspect of BR is not fully captured in terms of the dominance duration of each image and the alternation rate. The perception of brightness in BR (Levelt 1965, chap. 3) and the perceptual grouping in BR (Kovács *et al.* 1996; Alais *et al.* 2016; Stuit *et al.* 2017) have been studied. In addition, much attention has recently been attracted to *the transition phase* of the alternation between two images in BR (Wilson *et al.* 2001; Klink *et al.* 2013, sec. 2). It was found that the transition phase has two distinctive subjective features. First, the percept in BR changes “in *a wave-like fashion*, originating at one region of a figure and spreading from there throughout the rest of the figure” (Blake and Logothetis 2002, 3, emphasis added). Second, sometimes we see two images at the same time but at *different depths—*i.e. as if one figure is transparent and we are seeing the other figure through it (Yang et al. 1992).

Our experiment demonstrates that CEP is both a valid and productive method of inquiry. First, its results fit with the existing propositions about the subjective features of BR, in particular about *the influence of attention*. This consistency indicates the validity of CEP. Second, it deepens our understanding of *the spatiotemporal structure of the wave-like transition phase of BR*. Third, it elicits new research questions about the subjective features of BR, in particular about *the depth experience* in BR. The second and third contributions suggest the productivity of CEP. We shall conclude that CEP is a useful addition to the toolbox of experimental phenomenology, well suited to explore subjective features of experience that are difficult to capture by untrained introspective observation.

## Material and Methods

### Participants

Twenty healthy participants (age 20–28, mean = 23.3; 10 females) were recruited from the subject pool at Osaka University. They had normal or corrected-to-normal vision. None of them majored in philosophy (including Phenomenology as a philosophical discipline) or psychology. Although two participants reported that they had heard of BR, none of them experienced it first-hand before participating in the experiment. Informed consent was obtained from all the participants prior to the experiment. The experiment was conducted at the National Institute of Information and Communications Technology (NICT). The experimental protocol was approved by the ethics and safety committees of NICT.

### Stimuli

#### Apparatus

Visual stimuli were presented on a liquid-crystal display (Flexscan EV2780, Nanao, Japan) using PsychoPy (Peirce 2007) under Mac OS. The participants observed the stimuli from the distance of 90 cm between their eyes and the display through a mirror stereoscope (T.K.K.129, Takei Scientific Instruments Co. Ltd., Japan). The stereoscope was used to present the left half of the display exclusively to the left eye and the right half of the display exclusively to the right eye. For each participant, the eye holes and mirrors of the stereoscope were adjusted prior to the experiment so that the stimuli presented separately to both eyes were precisely fused.

#### Binocular rivalry

**Figure 1.**
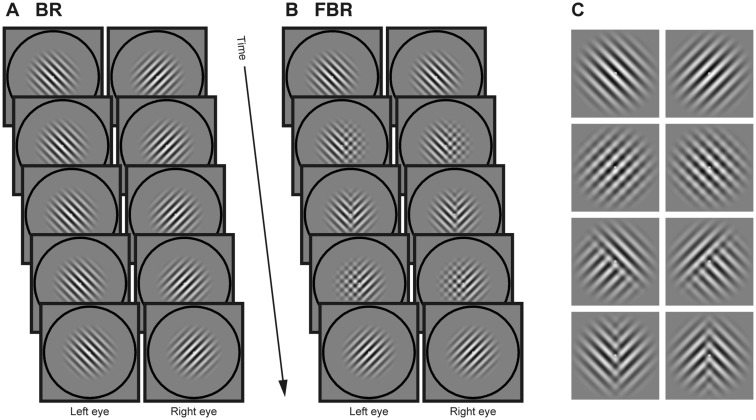
Stimuli. (**A**) In a BR trial, two Gabor patches with different orientations (BR stimuli) were dichoptically presented. Each patch was presented within a black ring frame. The participants viewed the patches while maintaining fixation to the green spot in the center of each patch. The BR stimuli induced illusional transformation of the figure. (**B**) In an FBR trial, the participants observed animations that mimic the transformation of the images induced by BR stimuli (FBR stimuli). In contrast to the BR stimuli, the FBR stimuli were presented binocularly. Also, in this case, each stimulus was enclosed by a black ring frame and had a centered green spot the participants fixated. The FBR stimuli induced actual transformation of the figure. (**C**) Eight reference patterns were used for generating the animations of FBR stimuli. The spatial patterns of transformations of FBR stimuli were produced by the random transition across the different reference patterns. The reference patterns were blending patterns of the two Gabor patches used in BR stimuli

BR stimuli consisted of Gabor patches [Fig. 1A; 1.7 cycles/degree of visual angle (c/dva), contrast = 100%]. Two patches were dichoptically presented on a gray background (34.5 cd/m^2^). Grating orientation differed between eyes (−45° and 45° from the vertical axis). Both patches were presented within a black ring frame (8.7 dva) to facilitate alignment of the eyes. Participants viewed the patches while maintaining fixation to a small green dot (0.2 dva) in the center of each patch. In addition to the fact that Gabor patches have been used extensively in existing BR studies (for instance, see Bonneh *et al.* 2001; Tsuchiya and Koch 2005; Brascamp and Blake 2012), there are two reasons for choosing gray-scale Gabor patches for our experimental research. First, we wanted the BR stimuli to be as simple as possible, because if they had more perceptible features such as hues or meaningful objects (e.g. house, face), the participants could focus more on such superficial features of the stimuli rather than subjective features distinctive of BR. Second, we wanted to induce BR-experiences with subjectively detectable transition phase, because the subjective aspect of transition phases of BR has been considered to be important for understanding dynamic neural processes underlying visual perception (Wilson *et al.* 2001; Knapen *et al.* 2011; Klink *et al.* 2013, sec. 2). The gray-scale Gabor patches satisfied these two requirements.

#### False binocular rivalry

False binocular rivalry (FBR) stimuli were animations that mimic the transformation of the image experienced when viewing BR stimuli (Fig. 1B). The animation was generated by the time-varying, position-dependent blending of the two Gabor patches composing the BR stimuli. While the BR stimuli were presented dichoptically and induced illusional transformation of the image (illusional in the sense that there is no figure that actually transforms), the FBR stimuli were presented binocularly and induced actual transformation of the figure by the animation. The other stimulus settings for the FBR stimuli were the same as used for the BR stimuli. Participants viewed the stimuli while maintaining fixation on the center dot.

FBR stimuli were designed to induce experiences that closely resemble genuine BR-experiences, which will allow us to produce phenomenal contrasts that can effectively illuminate the latter’s subjective features. In the experiment explained in Task procedure section, the participants were instructed to describe the subjective features of BR that were not present in FBR. Unless FBR is similar enough to BR, the comparison is not effective in revealing the distinctive features of BR that are difficult to notice by simple introspection. For instance, if FBR continued to transform at a constant speed, this would not be very helpful for the participants to explore the temporal features of BR in depth: they would simply notice the irregularity of the timings of transformations of BR, which is easily noticed by simple introspection without any contrasting experience. However, if FBR is designed to exhibit an irregular temporal pattern of transformation, then the participants are led to explore the experiential difference between BR and FBR more carefully and thereby capture subtler features of BR, such as how the transformation starts and ends, which are difficult to notice without the comparison.

The spatial patterns of transformations of FBR stimuli were produced by the random transition across eight different blending patterns of the two Gabor patches; those blending patterns were chosen to replicate the transition phase of BR (reference patterns; Fig. 1C). The first and second reference patterns were the original unblended patches used for the BR stimuli. The third and fourth reference patterns were the images in which the two patches were fully overlapped with each other: one was blended in the ratio of 1:4 and the other was blended in the ratio of 4:1. The fifth and sixth reference patterns were the images each of which consisted of the halves of the two patches conjoined with a blurred boundary; the boundary was tilted −45° or 45° from the vertical axis. The seventh and eighth reference patterns were the images in which the left half of one patch and the right half of the other patch were conjoined with a blurred boundary. The order of the transition from one pattern to another pattern was pseudo-randomly determined so as to yield all possible transitions with equal probability. In addition, different transition orders were used for each presentation of the FBR stimuli to the participants.

During individual transitions, the temporal patterns of the figure changes were designed to reproduce the dynamics of image transformation during BR. The time course of transitions from one reference pattern to another was generated using a recently developed computational model of BR (Li *et al.* 2017). This model simulates the dynamics of perceptual competition between two images in BR with attentional modulation and mutual inhibition (for details, see Supplementary methods). This model computes the response strength of two hypothetical neural populations corresponding to left- and right-eye perceptions from time to time. The time-varying response strength can be regarded as the time course of perceptual dominance of each rivalry image presented dichoptically. Although the original model was applied to the alternation between two rivalry images, the present study used the computed time course of perceptual dominance to determine the blending rate of the reference patterns during transitions. For example, at a given time point in the transition from a reference pattern A to a reference pattern B, if the model computed the dominance of 0.8 for a left-eye image and 0.2 for a right-eye image, the reference patterns A and B were blended in the ratio of 0.8:0.2 (=4:1). This procedure produced animations of figure transformation varying from time to time with various spatial patterns. Our codes for generating the FBR stimuli are available at https://github.com/Taiyou/FalseBinocularRivalryProject. The movie clip of the FBR stimuli is available at https://vimeo.com/375339505.

### Working hypothesis

Front-loading phenomenology is a recent strategy for incorporating the analytic perspective of philosophical phenomenology into experimental psychology (Gallagher 2003; Gallagher and Zahavi 2012). Phenomenology is a philosophical tradition founded in the beginning of the 20th century by Edmund Husserl and further developed by following philosophers, such as Martin Heidegger, Jean-Paul Sartre, and Maurice Merleau-Ponty. On a standard interpretation, its central characteristic lies in its distinctive interest in investigating conscious experience from the first-personal perspective of the conscious subject. To this end, the phenomenological method requires that studies of consciousness proceed by suspending or “bracketing” all theoretical and pre-theoretical assumptions concerning the metaphysical nature of consciousness (for more discussion, Gallagher and Zahavi 2012, 21–31; Smith 2018).

Recently, there is increasing interest in incorporating the methodology and the insights of this philosophical tradition into scientific approaches to mind and cognition (Gallagher and Zahavi 2012; Kaufer and Chemero 2015; Varela 2017). The notion of *front-loading phenomenology* has been proposed in this context as one way of achieving such interdisciplinary collaboration. Its basic idea is to use phenomenological reflection as a heuristic device for producing working hypotheses regarding psychological phenomena in designing experimental studies (Gallagher and Zahavi 2012, 44–45). This can be done either by drawing on phenomenological analyses developed by classical phenomenological philosophers or by attempting first-hand phenomenological reflections upon psychological phenomena of interest. The current experiment employed the latter approach: It started by formulating eight hypotheses concerning subjective features of BR based primarily on the experimenters’ first-hand experience of the phenomenon; the following experiment was designed to test these hypotheses against qualitative and quantitative data obtained through interview and questionnaire. This sub-section introduces the eight hypotheses, which are each labeled as follows: (i) *temporal indeterminacy*; (ii) *diachronic spatial indeterminacy*; (iii) *synchronic spatial indeterminacy*; (iv) *instability*; (v) *spatial disunity*; (vi) *sensitivity to subject’s change*; (viii) *ambiguous depth*; and (viii) *difficulty of fixation*.

#### Temporal indeterminacy

A major part of BR-experience consists of a dynamic transformation of the experienced content. As researchers of BR indicate, these transformations do not happen instantly “like successively exposed snapshots of one image and then the other” (Blake and Logothetis 2002, 3), but rather, they only happen through some stretch of time.

The first hypothesis concerns a subjective feature of the transition phase:

*Temporal indeterminacy*: Transformations in BR lack temporal determination.

Sometimes temporally extended events are perceived as something defined by a clearly determinate beginning and end. When you see a falling row of dominoes, e.g. you will see the moving pattern start at one point and finish at another point in time. Transformations of the percept in BR, however, lack specific start points or end points in time and hence fail to deliver a sense of temporal determinacy. This holds even when we pay careful attention to the whole transformation process including the experiences of each stable image *before and after the transition*. The transition phase of BR is typically described as “a wave-like intrusion of the alternative percept” (Klink *et al.* 2013, 311). Temporal indeterminacy can be counted as a characteristic of such wave-like transformations of BR.

#### Diachronic spatial indeterminacy

The second hypothesis indicates another feature of the transition phase of BR:

*Diachronic spatial indeterminacy*: Transformations in BR lack spatial determination.

Sometimes temporally extended events are perceived as having definite spatial properties, such as spatial trajectories and spatial boundaries. Once again, when seeing a falling row of dominoes, you will see the moving pattern start at a specific point and finish at another point in space. This hypothesis holds, however, that transformations in BR lack such determinate spatial properties and hence the transition phase of BR fails to deliver a sense of spatial determinacy; this feature is discussed in van Ee (2011). The diachronic spatial indeterminacy can also be counted as a characteristic of the wave-like transformations of BR.

#### Synchronic spatial indeterminacy

Each moment in the course of undergoing BR consists of a static experience of an image. The third hypothesis addresses a subjective feature of the static experience in BR:

*Synchronic spatial indeterminacy*: the content of static experiences in BR is spatially indeterminate.

Sometimes objects including 2D figures are perceived as having determinate spatial properties. For example, a coffee cup is typically experienced as having determinate spatial properties about its shape and location. This hypothesis states, however, that the content experienced at a particular moment in BR is spatially indeterminate. That is, a Gabor patch (or the mixture of two dissimilar Gabor patches) is experienced as being more obscure and indeterminate in BR compared with the experience of seeing it in non-BR conditions.

#### Instability

*Instability*: the content of BR-experience is prone to small and irregular changes so that it is experienced as being unstable.

Even during the relatively stable phases of BR-experience, it is prone to have small and irregular changes in its content. When an experienced content corresponds to either of the monocular stimuli, the BR-experience is very similar to the experience of seeing the stimulus in question with both eyes. Even in that stable phase, the content of BR continuously exhibits small changes in an irregular manner; it is sometimes experienced as being unstably vibrating. Instability may be related to the increase of microsaccades in BR (Sabrin and Kertesz 1980).

#### Spatial disunity

*Spatial disunity*: The alternation between two figures in BR is not always experienced as occurring on a single plane.

When seeing 2D figure transforming on a screen, the transformation appears to occur on a single plane. This hypothesis holds, in contrast, that the transformations in BR are not spatially unified in the same way. In the course of BR, e.g. there are phases in which the two figures corresponding to the two monocular stimuli are “both […] visible but appear to be located at different depth planes” (Yang et al. 1992, 47). It is as if one figure is transparent and we are seeing the other figure through it. The transformations and alternations in BR are sometimes experienced as being spatially disunified, as not occurring on the same single plane.

#### Sensitivity to subject’s change

*Sensitivity to subject’s change*: Transformations in BR are easily affected by the change of the subject’s conditions.

When seeing a 2D figure transforming, the patterns and timings of the transformation are not affected by the small changes of subject’s conditions such as attentional shifts and eye blinks. This hypothesis holds, in contrast, that the patterns and timings of transformations in BR are affected by such changes of the subject. This characteristic of BR has been widely discussed in BR studies (Ooi and He 1999; van Dam and van Ee 2005, 2006; Paffen and Van der Stigchel 2010; Hancock *et al.* 2012; Moreno-Sánchez *et al.* 2019).

#### Ambiguous depth

*Ambiguous depth*: The experienced depth of the figure is ambiguous in BR.

According to this hypothesis, the figures in BR are experienced as being out there, in front of our eyes, but without being in a particular distance from the viewpoint. This hypothesis can also be reasoned from the finding that there are phases in which the two figures corresponding to the two monocular stimuli are both visible but appear to be located at different depth planes (Yang et al. 1992, 47).

#### Difficulty of fixation

*Difficulty of fixation*: The experienced content of BR denies fixation.

While being presented with BR or FBR stimuli, the experimental participants are instructed to fixate on a fixation point located at the center of their visual field. But fixing one’s gaze on one point of the experiential content is more difficult in BR. If this proves to be an intersubjectively verifiable phenomenon, it may be seen as an experiential effect of a known fact about BR stimuli that they cause tiny eye movements and pupillary dilations not detected in non-BR conditions (Sabrin and Kertesz 1980; Blake *et al.* 2014, 6–7).

### Task procedure

**Figure 2.**
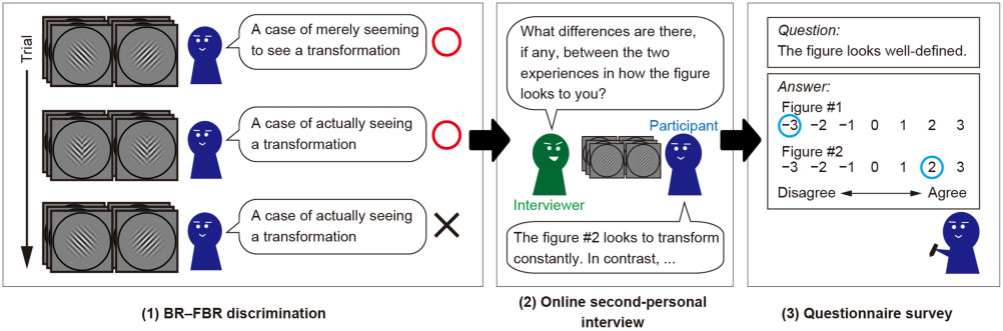
Experimental procedure. Our experiment consisted of three different parts. First, a BR–FBR discrimination task was performed to evaluate the discriminability of BR- and FBR-experiences. In this task, the participants were required to discriminate illusional transformations of the figure induced by BR stimuli and actual transformation of the figure induced by FBR stimuli on the basis of their own perceptual experiences. Second, an online second-personal interview was conducted to obtain detailed descriptions of BR-experience in contrast to FBR-experience. The participants reported their own experiences through the interaction with an interviewer while they viewed BR and FBR stimuli alternatingly. Finally, after finishing the viewing, the participants answered a questionnaire designed to examine our working hypotheses

The experiment consisted of three parts (Fig. 2). First, we instructed the participants to perform a BR-FBR discrimination task. Second, we asked them to describe the experiential difference between BR and FBR. Third, we conducted a questionnaire survey to the same participants to collect quantitative data concerning their experiences of BR and FBR.

#### BR–FBR discrimination

In the BR**–**FBR discrimination task, the participants were instructed to distinguish BR and FBR based on their experiences (Fig. 1C). On each trial, either BR or FBR stimuli were presented through the mirror stereoscope. The participants were required to verbally report whether they were actually seeing a transformation (FBR) or were seeing a mere appearance of a transformation (BR). Note that participants were just told that there are two kinds of experiences, one in which they see actual transformations occurring on the screen and another in which they see a mere appearance of transformations that do not actually occur on the screen; they did not have any further explanation as to what BR is. This design was intended to avoid the top-down biases on BR-experiences that may be caused by the participants’ expectations of what BR-experiences would be like (Lush *et al.* 2019). Immediately after the verbal report, the stimuli disappeared and the trial ended with no feedback about the correct answer. Although this means that each trial could last forever as long as the participants did not make a verbal report, we instructed them to make a verbal response as soon as they discriminated the stimuli and no participant spent an unreasonably large amount of time before making a response (for details of response time, see Task performance section). Across trials, the two stimuli were delivered in a pseudo-random order with equal probability (the participants were not informed of the fact that they have equal probability).

Every participant completed 10 successive trials of BR–FBR discrimination. If a participant made consistent judgments in the last five trials regarding the presented stimulus types (in the sense that two types of stimuli were consistently judged to be different regardless of whether the judgments are correct), the task was terminated after the 10th trial. Those who did not make consistent judgments in the last 5 trials went through the second round of 10 successive trials. If she made consistent judgments in the last five trials in the second round, the task was terminated there. Otherwise, the participant is sent to the final round, which we called the two-alternative discrimination, in which she was successively presented with a BR and an FBR stimulus and instructed to judge which was the case of actually seeing a transformation (FBR).

This discrimination task has three purposes. The first is to motivate the participants to concentrate on the subjective features of BR that are not present in FBR [since it is in general difficult for untrained people to properly focus on the subjective aspects of psychological phenomena (Miyahara et al. 2020)]. The second is to confirm that FBR is similar enough to BR to the extent that some participants sometimes mistake FBR for BR (see False binocular rivalry section). The third is to rule out the participants who lack the capacity to experientially discriminate BR from FBR (see Online second-personal interview section for the reason for this exclusion).

#### Online second-personal interview

In recent consciousness studies, second-personal interview has often been employed to elicit rich and reliable phenomenological reports from experimental participants who do not have relevant background knowledge (Petitmengin *et al.* 2007; Valenzuela Moguillansky *et al.* 2013; Høffding and Martiny 2016). We also adopted the second-personal interview to obtain descriptive data about BR- and FBR-experience. While there have been a few attempts to ask experimental participants to describe their experiences of BR (Cosmelli and Thompson 2007; Knapen *et al.* 2011), there is no attempt to conduct a systematic online second-personal interview for studying the subjective aspect of BR.

The interview consisted of three steps. During the whole interview, the participants were free to request the experimenter to present them with either the BR or FBR stimulus. This allowed them to describe their experience “online” without needing to recall past experiences.

In the first step, the participants were instructed to describe how they distinguished one type of experiences that they judged to be the case of seeing a mere appearance of a transformation (BR) and the other type of experiences that they judged to be the case of actually seeing a transformation (FBR). The reason for excluding the participants who fail the discrimination task is that this instruction works properly only for the participants who could correctly discriminate the experiences of BR and FBR. This question format allowed the participants to naturally focus primarily on the distinctive features of the two types of experience.

In the second step, they were followed-up by “content-empty questions” that only request clarifications of words or expressions that were used in the descriptions given in the first step (Petitmengin and Bitbol 2009, 385). For instance, we asked questions such as:

You stated that you had the impression that the transformation started from one side and gradually proceeded, could you explain this impression more concretely?

You stated that the experienced figure is a bit shaky and wobbly. Could you explain what you mean by this in more detail?

The purpose of introducing this method was to enrich the descriptive data without inducing biases.

In the third step, we conducted a “semi-structured interview” that explicitly targeted specific aspects of the experiences. In particular, we asked the participants to describe the differences between the two kinds of experiences, BR and FBR, in regard to *the way in which the figures look to them*, *the way in which they are looking at the figures*, and *the way in which they feel during the experiences*. The first two aspects roughly correspond to what Husserl analyzed as the “noematic” and “noetic” aspects of conscious experience (Gallagher and Zahavi 2012). The last aspect was added to allow the participants to note other features of their experience, which did not seem to answer the first two questions. When the answers to these questions were unclear or undetailed, once again, the participants were followed-up by content-empty questions for clarification.

#### Questionnaire survey

A questionnaire survey was also conducted to collect quantitative data about the subjective features of BR and FBR. The participants could not see each stimulus when answering the questionnaire. The participants filled in the questionnaire by writing. Except for the question 3, it used a scale of agreement from –3 to 3 (a seven-point Likert scale), which consisted of “strongly disagree” (–3), “disagree” (–2), “weakly disagree” (–1), “no opinion” (0), “weakly agree” (1), “agree” (2), and “strongly agree” (3). The questionnaire consisted of 25 questions (Table 1). Three questions of them (1–3) were designed to evaluate the difficulty of the discrimination task. The third question about the accuracy rate includes the options of 0–15%, 15–30% and 30–45% to cover the cases where participants consistently mistook BR for FBR in the discrimination task but noticed at some later point of the experiment that they made such mistakes. The remaining 22 questions (4–25) were designed to probe whether the working hypotheses applied to the participant’s experience. The participants were asked to respond to each question carefully by remembering the two kinds of experiences that they had during the discrimination task and the interview period.

**Table 1.** Questionnaire

Phenomenological hypothesis	Questionnaire items
	1. There was a clear difference between the two kinds of experiences that I had during the interview. 2. Everyone should be able to distinguish the two kinds of experiences that I had during the interview with 10 minutes of practice. 3. What do you estimate was the accuracy rate of your performance in the discrimination task? (If you took the discrimination task twice, please record your accuracy rate estimate of the second round.) Options: 0–15%, 15–30%, 30–45%, 45–60%, 60–75%, 75–90%, or 90–100%
Temporal indeterminacy	4. It was unclear when the transformation of the figure started and ended. 5. It was difficult to track the transformation of the figure consciously in real time. 6. Transformations of the figure started and ended unbeknownst to me such that I barely noticed that they have.
Diachronic spatial indeterminacy	7. The boundary between where and where not a transformation was going on was unclear. The shapes and directions of the transformation of the figure were obscure. 8. The shapes and directions of the transformation of the figure were obscure. 9. It was difficult to be consciously aware of where the transformation of the figure is taking place.
Synchronic spatial indeterminacy	10. The figure did not appear well-defined. 11. The figure looked well-defined.
Instability	12. The figure constantly alternated between transformation and stability. 13. The figure constantly continued to change in an unstable manner. 14. The figure changed in an unexpected manner.
Spatial disunity	15. It felt like multiple figures overlapped each other. 16. It felt like there were multiple figures not integrated very well within the same space. 17. The figure and the background were on the same plane.
Sensitivity to the Subject’s change	18. The experiential transformation of the figure occurred independently of how I directed my attention. 19. I could control the transformation of the figure. 20. How the figure transforms was felt to be determined in advance.
Ambiguous sense of distance	21. The distance to the figure always felt constant. 22. The distance to the figure felt obscure. 23. The distance to the figure was clear. 24. I had the impression that the figure moved slightly closer and further.
Difficulty of fixation	25. It was difficult to keep my gaze on the green fixation point.

### Data analysis

A phenomenological analysis was conducted of the descriptive data collected through the online second-personal interview, while statistical analysis was performed on the participants’ responses to the questionnaire.

#### Phenomenological analysis

The method of “descriptive phenomenological analysis” was adapted for this experiment to analyze the descriptive data. The method, also known as “the modified Husserlian approach” (Giorgi 2009), was developed by the phenomenological psychologist Amedeo Giorgi and is extensively used in the Duquesne School of phenomenological psychology. The analytic procedure consists of four stages (Langdridge 2007, 85–90):

The first stage is to read through each description to grasp its overall meaning. This stage is deemed important because different participants often use the same words to mean different things or different words to mean the same thing. Hence, what each part of the text means is determined not just by the lexical definitions of the words, but rather contextually in reference to the whole text.

The second stage is to break the descriptions down to distinct “units of meaning.” The goal of this stage is to attempt a parsing that is most relevant to the purpose of the experiment. In the current experiment, the primary task was to discern passages, phrases, and expressions that were more or less related to the working hypotheses. This does not mean that only the parts of descriptions that could be interpreted as *supporting* the working hypotheses were picked up; rather, the point was to tag every part of descriptions that could be relevant to the working hypotheses, including those that could potentially conflict with the working hypotheses.

The third stage is to assess the psychological significance of the meaning-units. In the current experiment, this was achieved indirectly by holding up each meaning-unit against the working hypotheses. In this stage, each meaning-unit was evaluated in regard to whether it supports/conflicts with each working hypothesis.

The fourth stage is to produce a general structural description of the experience. The orthodox approach is to generate structural accounts of the experience in question for each participant’s descriptive data and then to compare them to arrive at the most general structural account. In the current experiment, however, this last step was not taken because the purpose of the qualitative data analysis was not to develop a new account of the general structure of BR-experience, but rather to test the working hypotheses about it already generated through the front-loading method.

One final adaptation was that while Giorgi’s method is typically conducted by an individual analyst, we performed the analysis collectively. Each of the four authors conducted up to the first and second stages of analysis on five participants’ descriptions. After that, the examination of the validity of each parsing and assessment of the meaning-units were conducted through discussions among the four of us.

The examples of descriptions that can be counted as positive and negative evidence for each hypothesis were listed in advance. The examples helped us to carry out the whole phenomenological analysis (Hypotheses testing section).

#### Quantitative analysis

Statistical tests on the questionnaire scores were performed to quantify differences between BR- and FBR-experiences with regard to the phenomenological working hypotheses. Because seven of the eight hypotheses corresponded to multiple items in the questionnaire (Table 1), answers to these items were averaged within each hypothesis for each participant. Then, the scores for each hypothesis were compared between the BR and FBR conditions across participants. The correction for multiple comparisons was performed using false discovery rate (FDR; Benjamini and Hochberg 1995).

## Results

### Task performance

Twenty participants performed the BR–FBR discrimination task (Fig. 2). Each participant underwent up to three rounds of trials. The first two rounds consisted of 10 trials of BR–FBR discrimination, while the final round only included one trial (see Materials and Methods section). Nine out of 20 participants made consistent responses to the discrimination tasks in the first round. The rest of the 11 participants proceeded to the second round, out of which 6 participants gave consistent responses throughout the trials. The remaining five participants completed the two-alternative discrimination in the final round. No participant made consistent incorrect responses in the first round. One participant did so in the second round. In the final round, 1/5 participant failed to make the correct response. Since these two participants were regarded as unable to correctly discriminate BR- and FBR-experiences, the interview and questionnaire data from them were excluded from the data analyses.

For the participants who completed the task at the end of the first round, the mean correct rate was 88%, which was significantly higher than chance level (i.e. 50%; paired *t*-test, *P* < 0.0001). For the participants who completed the task at the end of second or third round, the mean correct rate in the first and second rounds was 70% and 83%, respectively, which were also significantly higher than chance (*P* < 0.0005); the rate tended to improve as the round proceeded although the improvement was not significant (paired *t*-test, *P* = 0.104). These indicate that almost all of the participants carried out BR–FBR discrimination with high accuracy, though they sometimes made a mistake in the discrimination task.

Overall, the response time on individual trials ranged between 1.6 and 55.2 (mean = 13.2) seconds. The response time averaged within each participant ranged between 6.9 and 21.1 (mean = 13.6) seconds. The mean response time differed neither between BR and FBR trials (mean = 14.8 vs. 12.3 s; paired *t*-test, *P* = 0.111) nor between when the participants felt that they were seeing BR and when they felt that they were seeing FBR (mean = 14.2 vs. 12.8 s; *P* = 0.261). These indicate that response time is unlikely to relate with the difference of BR- and FBR-experiences.

The questionnaire scores on the questions 1–3 (Table 2) also supported that the participants could discriminate the BR and FBR stimuli. The participants were agreeable to the question 1; their scores on the question were significantly higher than 0 (mean = 1.89; paired *t*-test, *P* < 0.0001). However, the scores on the question 2 indicate that the discrimination was not too easy for the participants; the scores were not significantly different from 0 (*P* = 0.355). In addition, the observation that the participants tended to underestimate their own task performance also indicates the difficulty of the discrimination; the estimated correct rate in the question 3 was significantly lower than the actual correct rate (*P* < 0.05). (However, this indication is not conclusive because the confidence rate about task performance may not correspond to the actual difficulty of the task. Note also that five participants chose either 0–15%, 15–30%, or 30–45% despite the fact that they did not explicitly behave like they started to suspect at some point of the experiment that they consistently mistook BR for FBR. This suggests that the participants misunderstood the statistical meaning of the question 3.)

**Table 2.** Response to each questionnaire item

Phenomenological hypothesis	Questionnaire items	Response (mean ± standard deviation [SD])	
	1	1.89 ± 1.57	
	2	0.5 ± 2.23	
	3	0.56 ± 1.65	
		BR	FBR
Temporal indeterminacy	4	1.61 ± 1.82	−1.1 ± 1.88
	5	1.17 ± 1.95	−1.7 ± 1.81
	6	1.00 ± 2.00	−1.3 ± 1.90
Diachronic spatial indeterminacy	7	1.72 ± 2.02	−1.9 ± 1.81
	8	−0.2 ± 2.34	−0.3 ± 2.33
	9	0.28 ± 2.22	−1.9 ± 1.55
Synchronic spatial indeterminacy	10	0.56 ± 2.38	−1.1 ± 2.17
	11	−0.2 ± 2.18	0.72 ± 2.32
Instability	12	−1.3 ± 1.56	1.44 ± 1.85
	13	0.67 ± 2.35	−0.5 ± 2.36
	14	−0.2 ± 2.07	0.72 ± 2.32
Spatial disunity	15	1.56 ± 1.85	−0.9 ± 2.17
	16	1.17 ± 1.89	−0.8 ± 2.16
	17	0.22 ± 1.93	1.33 ± 1.57
Attentional contingency	18	−1.1 ± 2.08	1.72 ± 1.84
	19	0.44 ± 2.23	−1.8 ± 1.59
	20	−1.5 ± 1.92	1.67 ± 2.03
Ambiguous sense of distance	21	1.0 ± 2.22	1.44 ± 1.65
	22	−0.3 ± 2.32	−1.5 ± 1.58
	23	−0.1 ± 1.97	1.22 ± 1.63
	24	−0.7 ± 1.88	−1.5 ± 1.62
Difficulty of fixation	25	0.0 ± 2.45	−1.2 ± 2.07

### Hypotheses testing

The working hypotheses were tested against the interview data (interview-based testing) and against the questionnaire data (questionnaire-based testing). The data from the two participants who could not correctly discriminate BR and FBR were excluded from the hypotheses testing.

The interview-based testing proceeded in three steps. First, we listed two categories of possible descriptive contents for each hypothesis: (i) Kinds of descriptions that the participants are expected to produce if each hypothesis is correct (*expected descriptions, ED*) and (ii) those that they are unlikely to produce under the same condition (*unexpected descriptions, UD*) (Table 3). Both categories included descriptive contents both about BR and FBR. Expected descriptions for BR were considered to be unexpected descriptions for FBR and *vice versa*. Second, each participant was tagged either as ED group (if they made only expected descriptions), UD group (if they made only unexpected descriptions), Neutral (if they made neither). No participant made both expected and unexpected descriptions for any given hypothesis. Finally, the hypotheses were evaluated based on these tags. A hypothesis was considered to be supported by the experiment if (i) none were tagged as UD group and (ii) more than 6 participants (one-third of the valid participants) were tagged as ED group. The two conditions were to justify that each working hypothesis is likely to hold for BR-experiences in general. Since there is no conventional standard to test a general hypothesis about a specific subjective feature of experiences based on the interview data, this standard was set out arbitrarily, but it is conservative enough to test the validity of the hypothesis.

**Table 3.** Expected (ED) and unexpected descriptions (UD)

Temporal indeterminacy	
ED	UD
It is unclear of the timing of the start/end of the transformation. It is difficult to attentively track how the image transforms. The transformation occurs before they were aware of it.	The transformation occurs right now. It is easy to track the process of the transformation. It is clear when the transformation occurred.
Diachronic spatial indeterminacy	
ED	UD
It is unclear where the transformations occurred. It is difficult to track how the figure transformed in shape and/or direction. It is unclear where the boundary between a changing area and an unchanging area is.	The transformation occurs at such and such place (concrete area/place). The shape/direction of the figure changes in such and such ways. A changing area is clearly distinguished from an unchanging area.
Synchronic spatial indeterminacy	
ED	UD
It is unclear what the figure looked like. The figure looks blurred/obscure. The boundary between patterns is obscure.	The figure looks like such. The figure looks clear (not blurred). The boundary between patterns is clear.
Instability	
ED	UD
The transformation occurs irregularly. The figure is vibrating unstably.	It is predictable when and how the figure transforms. The transformation occurs regularly. The figure is stable.
Spatial disunity	
ED	UD
Two figures are felt as overlapping. The fixation point is felt as disunified with the figure. The two patterns are not on the same plane.	One and the same figure transforms. The transformation occurs on a single plane.
Sensitivity to subject’s change	
ED	UD
The figure does not transform when concentrating on it. The timing of transformation correlates with attention shift.	The figure transforms on its own. The transformation does not depend on attention.
Ambiguous depth	
ED	UD
The distance to the figure is obscure/unclear. The distance to the figure is not always constant. The figure is felt as if it slightly got closer and went further.	The distance to the figure is unambiguous. The distance to the figure does not change.
Difficulty of fixation	
ED	UD
It is difficult to fix gaze on the fixation point. Effort is needed to fix gaze on the fixation point. Continuously gazing at the fixation point is tiring.	

**Figure 3.**
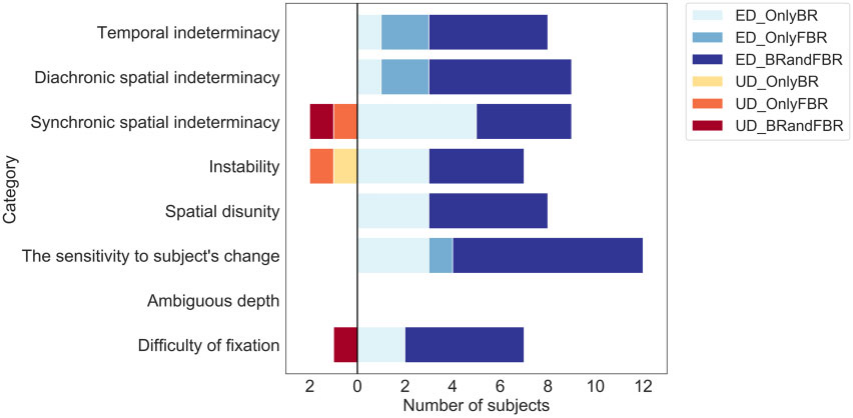
Interview-based hypotheses testing. Individual verbal reports of the participants in the interview were thoroughly assessed by a descriptive phenomenological analysis (see Materials and Methods section). Each color bar represents the number of the participants who provided ED or UD for each hypothesis regarding *BR and FBR*. Light blue, blue, and dark blue bars represent the number of the participants who provided ED only in BR, ED only in FBR, and ED in both BR and FBR, respectively. On the other hand, yellow, orange, and dark red bars represent UD only in BR, UD only in FBR, and UD in both BR and FBR, respectively

**Figure 4.**
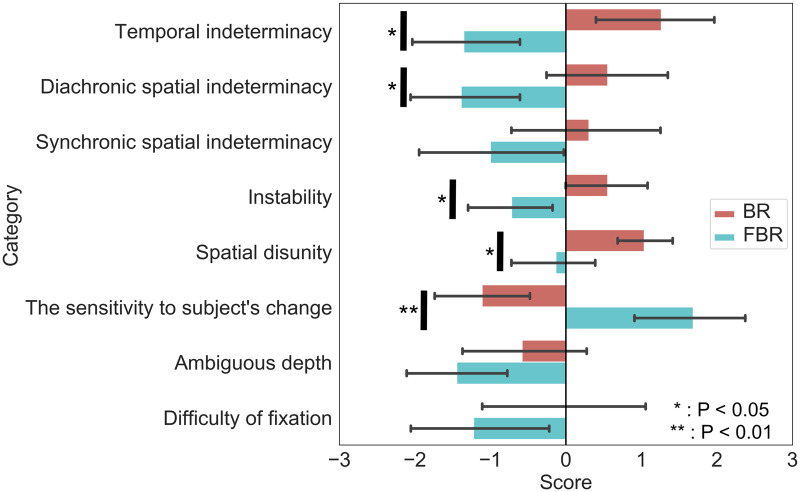
Questionnaire-based hypotheses testing. The scores in the questionnaire survey were compared between BR- and FBR-experiences. Each bar represents the mean score of BR (red) or FBR (cyan) experience regarding each of the phenomenological working hypotheses. Error bars denote the standard error of the mean (SEM). Asterisks indicate the scores significantly differed between the experiences (paired *t*-test with FDR correction; *P < 0.05; **P < 0.01)

The results of the interview testing are summarized in Fig. 3. The results of the questionnaire testing are summarized in Fig. 4. The following sections detail the results for each phenomenological hypothesis.

#### Temporal indeterminacy

The first hypothesis that *transformations in BR lack temporal determination* was supported by both the interview-based testing and the questionnaire-based testing.

- Interview-based testing: *Eight* participants were tagged as ED; no participant was tagged as UD. Here are two examples of ED:
  1-1 This one [BR], *I don’t get the timing* when the change occurs. [This] is like, I don’t get it, but *I find myself already seeing something different*.
  1-2 It’s not clear like [in BR], you’re not sure if it has changed or not, how can I put it, *it’s not a clear change like the second image [FBR], but you have a sense that it goes back and forth as it changes*. Um it’s like, not a clear change, so you have the impression that it hasn’t really changed.
  1-1 indicates that the participant could not identify the timing of the transformation. The phrase “I find myself already seeing” suggests that the start point in time was not registered in the experience. 1-2 shows that the participant felt the temporal trajectories of the transformations were unclear in BR. That it delivered the sense of “going back and forth” particularly suggests that when they started and ended were unclear to her. The description “so you have the impression that it hasn’t really changed” can be interpreted not as literally meaning that its transformation was not experienced but as just suggesting that it was the case of BR, considering the instruction of discrimination task, namely the instruction to verbally report whether they were actually seeing a transformation or were seeing a mere appearance of a transformation. The latter case can be described as the one in which the image does not really change.
- Questionnaire-based testing: The category-wise analysis exhibited statistical significance (*P* < 0.05, FDR-corrected paired *t*-test).

#### Diachronic spatial indeterminacy

The second hypothesis that *transformations in BR lack spatial determination* was supported by both the interview-based testing and the questionnaire-based testing.

- Interview-based testing: Nine participants were tagged as ED; no participant was tagged as UD. Here are two examples of ED:
  2-1 The lines change smoothly [in BR]. *It’s hard to tell where the change happened*. Once you think the whole image has the same kind of lines, but then a part of it changes its direction, but then once again all the lines are directed in the opposite or in a new direction, such that *I can’t describe it clearly like ‘it changed here in such and such a way’ as you see the image*.
  2-2 When each section changes separately [in FBR], I see the borders between the sections very clearly, and I’m like “Ah the border line lies here.”
  In 2-1, the phrase “it’s hard to tell where the change happened” clearly suggests that it was unclear where the transformation occurred. Likewise, the description of “I cannot describe it clearly like ‘it changed here in such and such a way’ as you see the image” suggests that it was unclear how the figure changed in shape and/or direction. The example 2-2 shows that it was *clear in FBR* where the boundary between a changing area and an unchanging area is, which suggests that it was unclear in BR.
- Questionnaire-based testing: The category-wise analysis showed statistical significance (*P* < 0.05, FDR-corrected paired *t*-test).

#### Synchronic spatial indeterminacy

The third hypothesis that *the content of static experiences in BR is spatially indeterminate* was neither adequately supported by the interview-based testing nor the questionnaire-based testing.

- Interview-based testing: Nine participants were tagged as ED, but two participants were tagged as UD. Here are two examples of ED.
  3-1 This one [BR] is more *like out of focus*.
  3-2 I think the distinctive feature [of BR] is that *it really feels like I’m seeing the same pattern a bit blurred all the way*. There’s a bit of a blur and the way it looks, it feels like as if I’m seeing a different pattern, as if the picture is unstable.
  In 3-1, the phrase “[BR] is more like out of focus” suggests that it looked blurred/obscure. In 3-2, likewise, the phrase “it really feels like I’m seeing the same pattern a bit blurred all the way” indicates that the figure looked blurred/obscure in BR.
  In contrast, here are two examples of UD:
  3-3 I have the impression that *the contours of the black lines are blurrier* in the tenth image [FBR].
  3-4 [In BR] It was like seeing black and white bands constantly, continuing, arranged diagonally. Sometimes I blink and it’s as if identical patterns intersecting with each other like a triangle, but *I have no impression that the image is blurred*.
  In 3-3, the phrase “the contours of the black lines are blurrier in [FBR]” suggests that the experienced figure looked clear in BR. In 3-4, the phrase “I have no impression that the image is blurred” suggests that the figure looked clear in BR.
- Questionnaire-based testing: The category-wise analysis showed no statistical significance.

#### Instability

The fourth hypothesis that *the content of BR-experience is prone to small and irregular changes so that it is experienced as being unstable* was supported by the questionnaire-based testing, but it was not supported by the interview-based testing.

- Interview-based testing: Seven participants were tagged as ED, but two participants were tagged as UD. Here are two examples of ED.
  4-1 *I get the impression that the whole image is vibrating* [in BR].
  4-2 *The way it looks changes irregularly and unsteadily* [in BR].
  4-1 clearly suggests that the figure was vibrating in BR. 4-2 indicates that the content of BR-experience changes irregularly and unsteadily.
  In contrast, here is an example of UD.
  4-3 *The direction of change is determined, or maybe predictable* [in BR], which makes it easier to see, but the tenth image [FBR] shows quite a few patterns, making it *harder to read which way it’s going to switch*, and I guess it is more thrilling because of this.
  The italic parts of 4-3 indicate that the content of FBR-experience changes more irregularly than that of BR-experience does. This is in conflict with the hypothesis of Instability.
- Questionnaire-based testing: The category-wise analysis showed statistical significance (*P* < 0.05, FDR-corrected paired *t*-test).

#### Spatial disunity

The fifth hypothesis that the alternation between two figures in BR is not always experienced as occurring on a single plane was supported by the interview-based testing and the questionnaire-based testing.

- Interview-based testing: Eight participants were tagged as ED; no participant was tagged as UD. Here are two examples of ED.
  5-1 In the ninth image [BR], every time I blink my eyes, it’s like, the diagonal orientations, these, *the diagonals descending from the right alternate between coming in front of and going behind the diagonals descending from the left*.
  5-2 The green point and the pattern are more unified [in FBR] than the ninth image [BR]. The ninth image looks like the green point is afloat.
  The italic parts of 5-1 suggest that the participant felt like two figures were overlapping in BR. 5-2 indicates that the participant felt like the fixation point was not unified with the figure in BR.
- Questionnaire-based testing: The category-wise analysis showed statistical significance (*P* < 0.05, FDR-corrected paired *t*-test).

#### Sensitivity to subject’s change

The sixth hypothesis that transformations in BR are affected by the change of the subject’s conditions was supported by the interview-based testing and the questionnaire-based testing.

- Interview-based testing: 12 participants were tagged as ED; no participant was tagged as UD. Here is an example of ED.
  6-1 [BR] changes its appearance slightly when I blink my eyes. The tenth one [FBR] […] appeared to change its pattern on the screen’s timing.
  6-2 [BR] It felt like perhaps the appearance is changing depending on how much effort I put to my eyes.
  6-1 clearly suggests that the timing of transformation depended on eye blinks. 6-2 also indicates that how the image looks in BR changes depending on the condition of eyes.
- Questionnaire-based testing: The category-wise analysis showed statistical significance (*P* < 0.05, FDR-corrected paired *t*-test).

#### Ambiguous depth

The seventh hypothesis that *the experienced depth of the figure is ambiguous in BR* is neither supported by the interview-based testing nor by the questionnaire-based testing.

- Interview-based testing: every participant was tagged as Neutral.
- Questionnaire-based testing: The category-wise analysis showed statistical insignificance.

#### Difficulty of fixation

The eighth hypothesis that *the experienced content of BR denies fixation* was not supported by the interview-based testing and also unsupported by the questionnaire-based testing.

- Interview-based testing: Seven participants were tagged as ED; one participant was tagged as UD. Here is an example of ED.
  8-1 It’s relatively easy to the green point as one [in FBR], but [in BR] the green point doesn’t appear as one unless I try to see it as one.
  Given that the “green point” means the fixation point, this description clearly suggests that the participant felt difficulty in fixing his/her gaze on the fixation point in BR. In contrast, one participant stated:
  8-2 Well I first keep my focus on the center, in the first one [BR], and I don’t get tired by keep seeing it, so it’s like I can keep seeing it, or better, I can see it with ease.
  This description suggests that the participant felt more tired by gazing at the fixation point in FBR.
- Questionnaire-based testing: There is no statistical significance.

## Discussion

The objective of this study was to introduce and justify CEP as a new experimental phenomenological method to investigate the subjective features of psychological phenomena. CEP proceeds in the following four steps: (i) *Front-loading phenomenology* (Working hypothesis section), (ii) *Online second-personal interview* (Online second-personal interview section), (iii) *Questionnaire survey* (Questionnaire survey section), and (iv) *Hypothesis-testing* (Hypotheses testing section). CEP employs *phenomenal contrast* in the experimental design, which distinguishes it from other experimental phenomenological methods, such as microphenomenology and experience sampling methods. We tested the validity and productivity of CEP by applying it to study the subjective features of BR. We first constructed eight hypotheses about the subjective features of BR and then tested them against two sets of phenomenological data obtained through online second-personal interview and questionnaire survey. Out of the eight hypotheses, four were supported by both the interview data and the questionnaire survey; one was only supported by the latter; three were supported by neither.

Among the four hypotheses which gained support from the experiment, *temporal indeterminacy* and *diachronic spatial indeterminacy* concern the transition phase of BR, which has attracted much attention in recent studies (Cosmelli and Thompson 2007; Klink *et al.* 2013, 310–311). Some earlier studies described this aspect of BR as exhibiting a wave-like transformation from one percept to another (Wilson *et al.* 2001; Blake and Logothetis 2002). Our findings offer a more specific understanding of this wave-like transition by clarifying that it is experienced as having both temporally and spatially indeterminate start and end points. This contribution demonstrates the productivity of CEP. We do not claim, however, that these features are universal among all forms of BR. As van Ee (2011) points out, BR stimuli can be designed to induce BR-experiences which involve transition phases with determinate spatial trajectories. They can also be designed to induce BR-experiences without any transition phase, which are in effect experienced as a sequence of switching between two percepts (Fox and Rasche 1969). Further empirical research is needed to determine the scope of these two hypotheses.

*Sensitivity to subject’s change* was also supported in the interview-based testing and the questionnaire-based testing. This finding provides additional evidence for the well-established proposition that the patterns and timings of transformations in BR are affected by changes in the subject, such as attentional shifts and eye movements (Ooi and He 1999; van Dam and van Ee 2005, 2006; Paffen and Van der Stigchel 2010; Hancock *et al.* 2012; Moreno-Sánchez *et al.* 2019). The consistency of our experimental result with this well-established phenomenological proposition strongly indicates the validity of CEP.

*Spatial disunity* was also supported both in the interview-based and the questionnaire-based testing. This indicates that the alternation between two figures in BR is not always experienced as occurring on a single plane. This provides additional evidence for the proposition that there are phases in which the two monocular stimuli are “both […] visible but appear to be located at different depth planes” (Yang et al. 1992, 47). Note, however, that *ambiguous depth* was neither supported in interview-based nor questionnaire-based testing. This is puzzling, because if there is a phase in which two figures appear to be located at different depth planes as if one figure is transparent and the other figure is seen through it, then the experienced depth of the figures is expected to be ambiguous. These results, in combination, give rise to novel research questions about the subjective aspect of depth perception in BR: what is the exact subjective character of the phase in which two figures appear to be located at different depth planes? How is depth experienced in BR? Is some level of expertise needed for one to be aware of the ambiguity of depth in BR-experience? Further phenomenological research is needed to address these research questions. The fact that CEP can produce such research questions, which will serve to move BR research forward in new directions, indicates its productivity as a method of inquiry.

*Synchronic spatial indeterminacy* and *Difficulty of fixation* were neither adequately supported in the interview-based testing nor the questionnaire-based testing. *Instability* was supported in the questionnaire-based testing but not in the interview-based testing. Nevertheless, these three hypotheses were not unanimously rejected in the interview-based testing: i.e. they all had a few participants lending supportive descriptions and hence categorized as ED group. This suggests the presence of individual differences in the relevant subjective aspects of BR: namely, how spatially indeterminate the contents of static experiences in BR are, how difficult it is to fix gaze on the fixation point in BR, and how much the content of BR-experience is prone to small and irregular changes. This interpretation of the results is indirectly supported by many findings in BR research showing that there are individual differences in BR-experience (Miller *et al.* 2003; Kanai *et al.* 2010; Yamashiro *et al.* 2014; Bosten *et al.* 2015). These results lead to new research questions: What is the prevalence of those who experience BR differently from the majority for each of these features? What are the neural, genetic, developmental, psychological, and behavioral factors responsible for these differences? Further research is required to address these research questions.

Finally, we conclude by making two methodological remarks on our research. First, as we saw above, we used *black-white Gabor patches* in our experiment, and it remains unclear to what extent we can generalize our findings in this study to BRs induced by other kinds of BR stimuli. In fact, no BR stimuli would allow us to establish general claims about the phenomenon. Notice, however, that this is not so much an in-principle problem with CEP as simply indicating a healthy open-endedness of the study: Just as any other form of scientific investigation, experimental phenomenology can approach the essential features of psychological phenomena only through the constant effort of accumulating new data to test and update existing hypotheses.

Second, the results of our experiment also depend on the specific design of FBR stimulus. There are other ways to design FBR stimuli. For instance, some researchers such as Knapen *et al.* (2011) have used duration-matched replays as FBR stimuli. Duration-matched replays are designed individually based on each participant’s report on the dominance duration of each stimulus and the timing and length of the transition phase in their actual experience of BR, so the replay exhibits the same dynamic profile as that reported during actual rivalry. If we used duration-matched replays to obtain a phenomenal contrast with BR, the participants might have focused on different subjective features. Furthermore, if the FBR resembled the BR to the extent that they are subjectively indiscriminable, we could not collect any description of BR in the framework of CEP. However, this does not indicate that the descriptions acquired by using our FBR stimulus did not correctly capture the subjective aspect of BR. Data collection in CEP certainly depends on the nature of the phenomenal contrast that has been used, and so in this case on the character of the FBR stimulus, but this is not an essential difficulty of the methodology itself. One analogy may be useful to clarify this point. When we try to describe the flavor of *vegemite* in detail, it is helpful to compare it with other fermented salty food. One can become aware of different aspects of the vegemite flavor by comparing it with several different kinds of food, such as Japanese salted fish guts and Miso. Descriptions of vegemite acquired through comparisons with these foods are not invalidated by the existence of *marmite*, whose flavor is subjectively indiscriminable from vegemite. Likewise, if two kinds of FBR stimuli differ in some experiential aspects (as in ours and duration-matched replays), one would be aware of different subjective features of BR through comparing it with each kind of FBR. The existence of perfect FBR subjectively indiscriminable from BR, however, does not invalidate descriptions of BR obtained through comparisons with these imperfect FBRs. We can employ different kinds of FBRs for further research, and this is exactly one of the ways to tackle the research questions presented above.

We have argued for the validity and productivity of CEP based on our study of BR employing this proposed method. In principle, CEP can be applied to any psychological phenomenon that experimental psychology addresses. One particularly fruitful area of application is virtual reality: CEP may be used to explore and specify the subjective difference between the experience of virtual reality and non-virtual reality environments; this should help researchers develop virtual reality technologies that can produce a more genuine sense of reality. No doubt, there is much room for improvement in our proposed version of CEP. Yet we suggest that CEP offers a promising experimental schema to explore elusive subjective features of psychological phenomena that are difficult to capture by untrained introspective observation.

## Supplementary data

Supplementary data are available at *NCONSC Journal* online.

## Author Contributions

T.N., M.K., H.T.H., and S.N. designed the research; T.N. and S.N. performed experiments; T.N., M.K., H.T.H., and S.N. conducted the data analyses and wrote the manuscript.

## Supplementary Material

niaa018_Supplementary_Data
