# Supplementary material for: A new experimental phenomenological method to explore the subjective features of psychological phenomena: its application to binocular rivalry
Source: Neurosci Conscious. 2020 Oct 3;2020(1):niaa018. doi: 10.1093/nc/niaa018 (PMC7532693; doi:10.1093/nc/niaa018)
Supplement: niaa018_Supplementary_Data [file niaa018_supplementary_data.docx]

**The Supplementary Methods:**

A recently developed computational model was used to mimic the time course of perceptual transitions during BR (Li et al., 2017). The model was originally introduced to demonstrate the role of visual attention in modulating perceptual competition in BR, but it captures the corresponding dynamics of neuronal population activity in the visual cortex well. The kind of BRs addressed in these studies was those induced when two visual stimuli in orthogonal orientations were presented dichoptically.

The model is constituted by three classes of units respectively involved in sensory representation, mutual inhibition, and attentional modulation. The response of each individual unit simulates the mean activity of the neuronal population with similar response properties. The units responsible for sensory representation include monocular and binocular-summation units. The monocular units capture the transition of sensory representations for each eye and each orientation. The excitatory and inhibitory drives to the monocular units are determined by the combination of external sensory inputs, response adaptation, and the competition between opponency units through mutual inhibition and attentional modulation. The binocular-summation units receive signals from the monocular units and represent the perceptual transition between dichoptic images after the monocular-unit representations are integrated. The units responsible for mutual inhibition and attentional modulation receive signals from the sensory-representation units and modify the response of the monocular units. Mutual inhibition emphasizes one of the two eye representations by suppressing another representation. Attentional modulation increases the contrast gain between the two orientation representations. The step-by-step calculation of this model generates the time series of unit responses.

Our simulation with this model was performed in MATLAB codes implemented by the researchers who developed the original model (Li et al., 2017; http://hdl.handle.net/2451/38721), using their default setting of model parameters. The time series of responses in the binocular-summation unit were used to generate the temporal pattern of figure changes in FBR stimuli. The time series have positive and negative peaks that are alternately repeated. One of the eight different reference patterns (Figure 1C) was pseudo-randomly allocated to each of these positive and negative peaks. Then, in each transition between the peaks, the blending ratio of the two reference patterns at a given time point was determined by the response strength of the binocular-summation unit at the same time point. However, the transition generated from the original model seemed to be too slow and had no stable periods, which evidently contradicted our observation of BR during the preparation of the experiment. Hence, the transition speed was increased by 10 times, and a period of 0–5 seconds during which the figure made no change was appended to each peak by drawing the duration from a uniform probability distribution. Following this procedure, five different temporal patterns of figure changes in FBR stimuli were produced for the main experiment.
